# Supplementary figures and images for: A reliable and inclusive method for assessing failure-causing mechanical wear in prosthetic feet
Source: PLOS Glob Public Health. 2026 May 29;6(5):e0006545. doi: 10.1371/journal.pgph.0006545 (PMC13221059; doi:10.1371/journal.pgph.0006545)

# Ranson et al., 2023 (Table 1)

**3 raters, 62 feet**

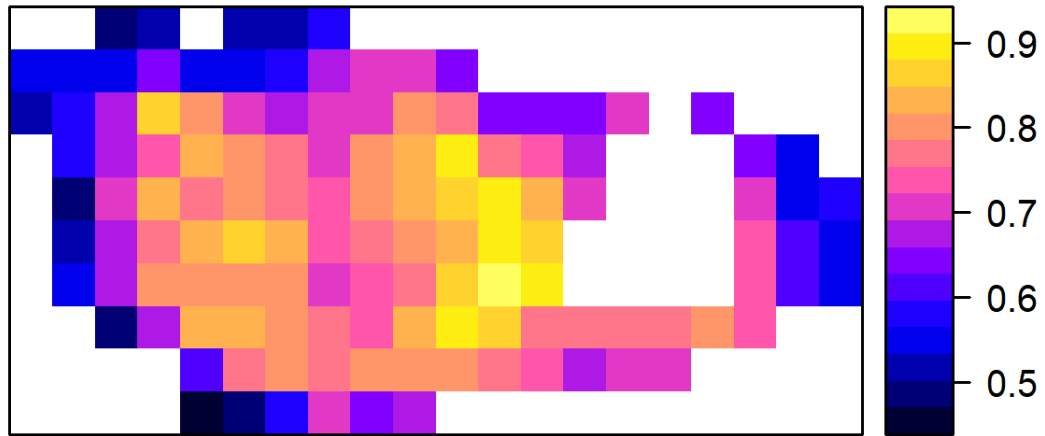

**4 raters, 48 feet**

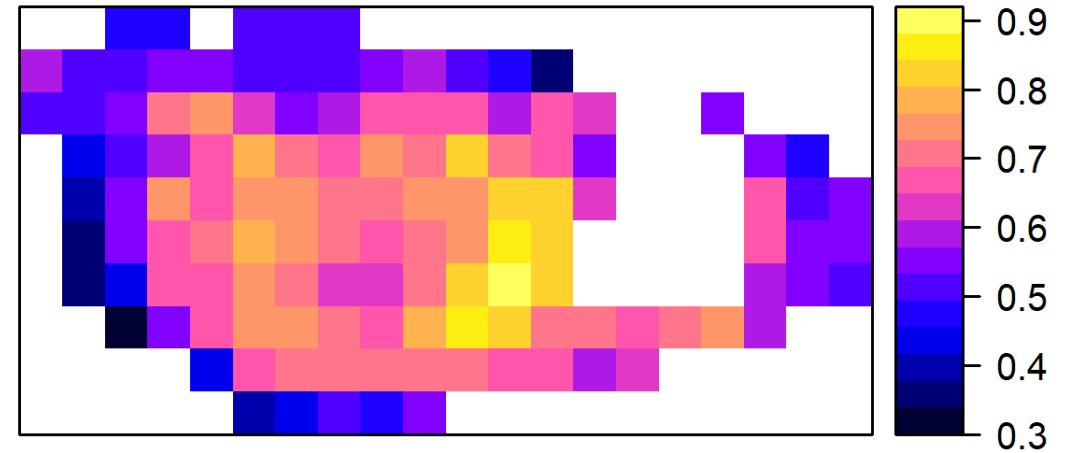

# Modified Ranson et al., 2023 (Table 2)

**3 raters, 62 feet**

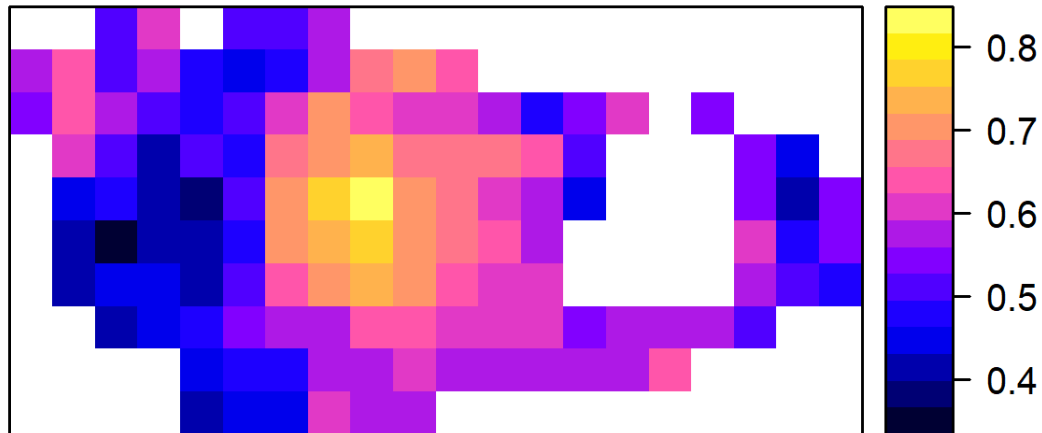

**4 raters, 48 feet**

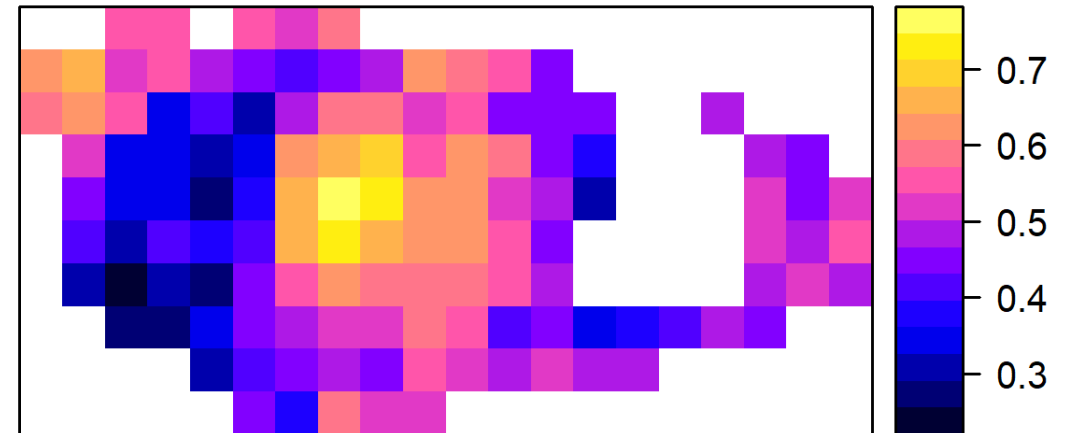

Supplement: S1 Fig — (PDF) [file pgph.0006545.s001.pdf]
