# Supplementary material for: A reliable and inclusive method for assessing failure-causing mechanical wear in prosthetic feet
Source: PLOS Glob Public Health. 2026 May 29;6(5):e0006545. doi: 10.1371/journal.pgph.0006545 (PMC13221059; doi:10.1371/journal.pgph.0006545)
Supplement: Attachment — Submitted filename: Response to reviewers.docx [file pgph.0006545.s006.docx]

1. Please amend your detailed Financial Disclosure statement. This is published with the article. It must therefore be completed in full sentences and contain the exact wording you wish to be published.

i. Please clarify all sources of financial support for your study. List the grants, grant numbers, and organizations that funded your study, including funding received from your institution. Please note that suppliers of material support, including research materials, should be recognized in the Acknowledgements section rather than in the Financial Disclosure. 
ii. State the initials, alongside each funding source, of each author to receive each grant. For example: "This work was supported by the National Institutes of Health (####### to AM; ###### to CJ) and the National Science Foundation (###### to AM)."
iii. State what role the funders took in the study. If the funders had no role in your study, please state: “The funders had no role in study design, data collection and analysis, decision to publish, or preparation of the manuscript.”
iv. If any authors received a salary from any of your funders, please state which authors and which funders.

We have amended this section to add appropriate funding information. Please note there were no grants from In2STEM or JJCDR, but they provided in-kind support (In2STEM, recruiting students and linking them to academics; JJCDR, collecting feet). We felt this belonged in the Financial Disclosure section, as it was in-kind financial support, covering salaries, overheads, etc. If it does not, we are happy to move where it belongs – please just let us know.

2. Please send a completed 'Competing Interests' statement, including any COIs declared by your co-authors. If you have no competing interests to declare, please state "The authors have declared that no competing interests exist". Otherwise please declare all competing interests beginning with the statement "I have read the journal's policy and the authors of this manuscript have the following competing interests:"

Amended

3. Please note that your Data Availability Statement is currently missing the repository name and/or the DOI/accession number of each dataset OR a direct link to access each database. If your manuscript is accepted for publication, you will be asked to provide these details on a very short timeline. We therefore suggest that you provide this information now, though we will not hold up the peer review process if you are unable.

All data is supplied in supplementary material, as stated in the data availability section.

4. We noticed that you used “unpublished data" in the manuscript. We do not allow these references, as the PLOS data access policy requires that all data be either published with the manuscript or made available in a publicly accessible database. Please amend the supplementary material to include the referenced data or remove the references.

We do not use unpublished data. We have a sentence in the introduction where we state observations, then say they are unpublished. If this is not acceptable, please let us know and we can remove the sentence.

5. We have noticed that you have uploaded Supporting Information files, but you have not included a list of legends. Please add a full list of legends for your Supporting Information files after the references list.

Added

If the reviewer comments include a recommendation to cite specific previously published works, please review and evaluate these publications to determine whether they are relevant and should be cited. There is no requirement to cite these works unless the editor has indicated otherwise. 

**Additional Editor Comments (if provided):**

Thank you for this submission. The editorial team is in agreement with the documentation of a reviewer that this manuscript addresses quantifying wear-related failure in prosthetic feet, which is under explored in low-resource settings.

The manuscript submitted still has a few comments and unapplied references, giving the impression of a work in progress or unfinished version being submitted.

We sincerely apologise for this error – we accidentally uploaded a copy of the manuscript which was not fully formatted. We can assure the editor the content of the manuscript was accurate and up to date.

To reiterate very serious concerns raised, there are challenges seen in the following care aspects of the study that may prevent publication.

1) the study’s methodological rigor

We have taken these factors into account and addressed below. But briefly have made some comments here.

- a focus exclusively on inter- and intraobserver reliability, without establishing validity.
- no comparison with any objective or quantitative measure of wear,

As explained below, this is not needed, and the method we are employing has already gone through peer-review and is an accepted method within the community.

- difficult to determine whether the scoring system reflects actual material degradation.

It does not representing material degradation but material removal. It is based on quantifying how much of the material is left, and therefore what has been removed.

- Study design- the use of untrained student raters is framed as a robustness test, but in practice, it introduces substantial variability and protocol deviations (including one group failing to correctly apply the method). This erodes the method’s usability and reproducibility.

We believe that the fact that the test showed robust results, despite this substantial variability and protocol deviations, demonstrates the robustness of the method. I.e., if we can get good results even when this variability is present, the method is truly highly robust.

- intraobserver reliability is assessed using a very small subset (two raters, five images)- insufficient to draw strong conclusions.
- reliance on Kendall’s W (which is sensitive to ties), the lack of complementary agreement metrics, and aggregating raters into groups, which may mask within-group variability.
- The image-based scoring approach is not standardized (e.g., lighting, angle), which could further affect reproducibility.

2) the strength of its conclusions.
As a result of methodological loopholes, the conclusions, particularly those suggesting clinical applicability, appear overstated.

Challenges to External validity and generalizability (a single center in Sri Lanka, primarily of similar SACH-type construction), but claims are much more generalized.

Please reframe this as a preliminary reliability assessment rather than a fully validated method. While it has potential, substantial revision is needed to appropriately frame the contribution, temper the claims, and more clearly acknowledge limitations.

The intraobserver error results have been reframed as preliminary, but the interobserver reliability results are not preliminary. The method is not generally applicable to all feet and is therefore not the “end-all-be-all” for prosthetic foot wear methodologies, but this paper is about the reliability of the method, and not its development.

Kindly address each review comment in detail and apply changes to proceed.

We have addressed these concerns in the manuscript and responded below.

[Note: HTML markup is below. Please do not edit.]

Reviewers' comments:

Reviewer's Responses to Questions

**Comments to the Author**

**Reviewer #1:** Dear authors, the manuscript seems to be not finished, please resubmit when it is fully finished and revised. In the current form there are annotations and not completed references, before submitting everithing should be double checked.

We sincerely apologise for this error – we accidentally uploaded a copy of the manuscript which was not fully formatted. We can assure the reviewer the content of the manuscript was accurate and up to date.

**Reviewer #2:** This manuscript addresses an important and underexplored issue: quantifying wear-related failure in prosthetic feet, particularly in low-resource settings. The topic aligns with the journal’s scope, and the concept of a low-cost, field-applicable wear assessment tool is valuable. However, several concerns arise regarding the study’s methodological rigor and the strength of its conclusions.

We thank the reviewer for taking the time to provide their valuable feedback on this manuscript.

The primary issue is that the manuscript focuses exclusively on inter- and intraobserver reliability, without establishing validity. There is no comparison with any objective or quantitative measure of wear, which makes it difficult to determine whether the scoring system reflects actual material degradation. Consequently, the conclusions, particularly those suggesting clinical applicability, appear overstated.

We are unsure what the review means by “without establishing validity”. There is no need to perform objective or quantitative measures of wear (e.g., measuring volume or mass of prosthetic foot lost), and then correlating it to the scoring method, and hopefully this response will help explain why.

1) **Study aim** – if the study aim was, e.g., to understand the mechanisms or rate of wear, we agree we would need to know things like wear rates. However, this was not the aim of the study. Rather, the aim is to investigate the reliability of a published method for quantifying wear patterns on feet.
2) **A valid method of quantifying wear** – we have employed knowledge of the manufacture of the prosthetic feet used in this study to validate the method. The manufacture of the feet is described in detail in Ranson et al., 2024. Briefly, two nylon layers are placed on the bottom of the foot and held in place with natural rubber. The exposure of the first, then the second layer of nylon therefore represents removal of material from the plantar surface of the foot. While it is possible small amounts of material may be removed by, e.g., chipping of small pieces of rubber, or pull out of fibres, we have not observed this. Occasionally, feet which are manufactured using nylon without fibres (not used here) exhibit delamination and this leads to material loss, but we have not seen this in any of the ~700 feet we currently have in the collection with fibres on the plantar surface.

We have added the following section to the M&M.

## Validation of the wear scoring method

Methods for quantifying wear usually rely on quantifying, e.g., changes in mass or volume over time, which is useful for understanding how to predict wear over time. While useful for design and failure analyses, it is not needed here, where we are quantifying patterns and magnitude of wear on the plantar surface of the foot.

The design and manufacture of the feet used in this study is described in [7]. Briefly, two layers of fibre reinforced nylon sheets are placed on the bottom of a foot mould with natural rubber to create the plantar surface of the foot. Therefore, the exposure of the first, then the second, layer of nylon represents removal of material from the plantar surface, and the exposure of the second layer represents more material removal than the exposure of the first layer. This makes the method being investigated here specific to this design of prosthetic foot, and we have begun to investigate how to generalize this method for other designs of prosthetic feet (below).

The study design also raises concerns. The use of untrained student raters is framed as a robustness test, but in practice, it introduces substantial variability and protocol deviations (including one group failing to correctly apply the method). This undermines confidence in the method’s usability and reproducibility, especially in real-world settings where consistent training may also be limited.

We argue the opposite – the collaboration with these students bolsters the method’s usability and reproducibility, particularly in real-world settings where consistent training may also be limited. Student raters with limited training (not untrained, as we ran a training session – please see “training” subsection in the M&M) were likely to introduce substantial variability. However, we have been able to demonstrate that, despite this, the results are relatively robust. This means that – if/when applied in real-world settings where consistent training may also be limited – the method is likely to be robust and produce reliable results. If we had highly trained e.g., prosthetists or wear specialists employing this method, it would create “best case” results here, and it would be likely usability and reproducibility would be lower in real-world settings. Our study therefore produces results that are more likely to mimic those seen in real-world settings.

We have added this section

To maximize the chances of interobserver error, and truly examine the reliability of this method, teenage students from local schools, who lacked backgrounds in prosthetics and therefore true novices (SQ, RO, TM, RH, NA, SA), were recruited for this study. The collaboration with novices is likely to introduce substantial variability, which likely mimics or exceeds the variability that would be observed in real-world (e.g., clinical) settings, where consistent training may be limited. These results therefore represent true “worst case” reliability scenarios and – if the method is reliable and robust in this setting – it will likely be reliable to be used in real-world settings.

Additionally, intraobserver reliability is assessed using a very small subset (two raters, five images), which is insufficient to draw strong conclusions.

We have tempered the language throughout

External validity is another limitation. The dataset is restricted to prosthetic feet from a single center in Sri Lanka, primarily of similar SACH-type construction. This significantly limits the generalisability of the findings, yet the manuscript makes relatively broad claims about applicability across settings and prosthetic designs.

We have added a section to limitations and altered the abstract to reflect this.

There are also statistical and methodological concerns, including reliance on Kendall’s W (which is sensitive to ties), the lack of complementary agreement metrics, and aggregating raters into groups, which may mask within-group variability. The image-based scoring approach is not standardized (e.g., lighting, angle), which could further affect reproducibility.

Kendall’s W is the appropriate test statistic to use here. We discussed how limitations of the method affected our results, but as the reviewer has not suggested additional tests, no further statistics were performed.

We are unsure what is meant by “lack of complementary agreement metrics”. If the reviewer can state what metrics/statistics they wish to see, we would be happy to include them.

Raters were not aggregated into groups but rather worked in groups. There are no individual marks that were aggregated. We statistically treated groups as individual raters, which is appropriate as raters do not need to be distinct individuals and can be distinct groups.

The scoring approach is standardized (see Ranson et al., 2024). Photographs were standardized in terms of angle (foot position – the plantar surface of the foot was photographed, e.g., “… photograph of the plantar surface of each foot …”, “…pictures of the plantar surfaces of the feet.” While slight angles of the plantar foot relative to the camera may affect grid placement, and therefore cell homology, other factors would not affect the results here (e.g., was rubber missing, nylon present/absent). An investigation into the robusticity of this method to photography methods is certainly valuable, but beyond the scope of this work.

Overall, I view this study as a preliminary reliability assessment rather than a fully validated method. While it has potential, substantial revision is needed to appropriately frame the contribution, temper the claims, and more clearly acknowledge limitations.

We thank the reviewer for their conclusions. While the method is fully validated (see above) and the interobserver error analysis is complete, the intraobserver results should be viewed as preliminary and we have altered the language to reflect this.
